# To assess the effectiveness of various communication strategies for improving childhood pneumonia case management: study protocol of a community based behavioral open labeled trial in rural Lucknow, Uttar Pradesh, India

**DOI:** 10.1186/s12887-018-1250-4

**Published:** 2018-08-22

**Authors:** Shally Awasthi, Tuhina Verma, Monica Agarwal, Chandra Mani Pandey

**Affiliations:** 1 Department of Pediatrics, King George’s Medical University, Lucknow, India; 2 Department of Community Medicine, King George’s Medical University, Lucknow, India; 3 Departmentof Biostatistics and Health Informatics, Sanjay Gandhi Postgraduate, Institute of Medical Sciences, Lucknow, Uttar Pradesh India

**Keywords:** Community acquired pneumonia, Under 5, Behavior change, Trial, Quality of care

## Abstract

**Background:**

Community Acquired Pneumonia (CAP) is the leading cause of childhood morbidity and mortality worldwide including India. Many of these deaths can be averted by creating awareness in community about early symptoms of CAP and by ensuring availability of round the clock, quality health care.

The objective was to assess the effectiveness of an innovative package of orienting doctors and community health workers about community perceptions on CAP barriers to qualified health care seeking, plus infrastructural strengthening by (i) providing “Pneumonia Drug Kit” (PDK) (ii) establishing “Pneumonia Management Corner” (PMC) at additional primary health center (PHCs) and (iii) “Pneumonia Management Unit” (PMU) at Community health center (CHCs) along with one of 4 different behavior change communication interventions**:**

1. Organizing Childhood Pneumonia Awareness Sessions (PAS) for caregivers of children < 5 years of age during a routine immunization day at PHCs and CHCs by Auxillary Nurse Midwives (ANM)
2. Organizing PAS on Village Health and Nutrition Day only once a month in villages by Accredited Social Health Activist (ASHA)
3. Combination of both Interventions 1 & 2
4. Usual Care

as measured by number of clinical pneumonia cases-treated by ANM/doctors with PDK or treated at either PMC or PMU.

**Methods:**

Prospective community based open labeled behavioral trial (2 by 2 factorial design) conducted in 8 rural blocks of Lucknow district. Community survey will be done by multistage cluster sampling to collect information on changes in types of health care providers’ service utilization for ARI/CAP pre and post intervention.

**Discussion:**

CAP is one of the leading killers of childhood deaths worldwide. Studies have reported that recognition of pneumonia and its danger signs is poor among caregivers. The proposed study will assess effectiveness of various communication strategies for improving childhood pneumonia case management interventions at mother/community level, health worker and health center level. The project will generate demand and improve supply of quality of care of CAP and thus result in reduced mortality in Lucknow district. Since the work will be done in partnership with government, it can be scaled up.

**Trial registration:**

This study has been registered retrospectively in the AEARCT Registry and the registration number is: AEARCTR-0003137.

**Electronic supplementary material:**

The online version of this article (10.1186/s12887-018-1250-4) contains supplementary material, which is available to authorized users.

## Background

Pneumonia, the leading cause of childhood morbidity and mortality worldwide, is responsible for deaths of more than 2 million children annually [1]. Among these, two-third deaths are concentrated in just 10 developing countries, India being one of them. It is estimated that 408,000 children less than 5 years die due to clinical pneumonia in India [2]. Many of these deaths can be averted by creating awareness of the community about early signs of pneumonia and by ensuring availability of round the clock, quality health care.

After extensive formative research on childhood pneumonia in Uttar Pradesh and Bihar (14 districts), we found that pneumonia related morbidity and mortality can be averted if the following barriers are addressed: (a) delay in symptom recognition (b) delay in timely and qualified health care seeking (c) distrust of the community on the available public health services [3]. Thereafter, we developed and validated text, audio, video messages to address these barriers [4]. Specifically, messages were developed on (a) symptom recognition (b) where and when to seek treatment (c) how to approach a care provider and negotiate for quality of care (d) risk vulnerability perception. The proposed project aims to leverage the extensive work done and conduct operations research to address these three barriers to health care seeking through innovative community based approaches using messages developed by us as well as by strengthening the existing public health system.

At present, only 70.8% rural children seek care for symptoms of acute respiratory infections [5]. Our hypothesis is that strengthening of public health system to provide sustainable quality care for cases of childhood pneumonia (CAP) followed by strategic dissemination of validated messages to community by public health grass-root  workers may improve care seeking behavior for CAP within 12 months from qualified public health care providers. Goal of this project was to enhance early recognition and care seeking for CAP from public health system by ensuring empowerment of community and care providers for delivery of round the clock, quality-care.

## Methods/design

### Study aim

Our primary aim is to assess the effectiveness of an innovative package of orienting doctors and community health workers (CHW) about community perceptions on CAP barriers to qualified health care seeking plus infrastructural strengthening by (i) providing “Pneumonia Drug Kit” (PDK) (ii) establishing “Pneumonia Management Corner” (PMC) at additional primary health center (APHC) and (iii) “Pneumonia Management Unit” (PMU) at Community health center (CHC) *ALONG* with one of the 4 different behavior change communication (BCC) interventions:

- Intervention 1: Organizing Childhood Pneumonia Awareness Sessions (PAS) for caregivers of children < 5 years of age during a routine immunization day, using self-developed and validated Information, Education and Communication (IEC) materials, in PHCs and CHC monthly, conducted by a trained Auxillary Nurse Midwife (ANM) and project facilitators.
- Intervention 2: Organizing PAS on Village Health and Nutrition Day (V.H.N.D.) once a month by the Accredited Social Health Activist (ASHA) trained to conduct such sessions
- Intervention 3: Combination of Both Intervention 1& 2
- Intervention 4: Usual Care

Outcome measure will be number of CAP treated by ANMs/doctors with medicines from PDK or treated at either PMC or PMU in interventions given by ANMs/ASHA workers.

Our second objective is to ascertain change, if any, in the types of health care providers’ service utilization for Acute Respiratory Illness (ARI)/CAP in last 12 months in children less than 5 years pre and post intervention.

Outcome measure will be number of ARI/CAP Treated by ANMs/doctors by various health care providers in the past 12 months in interventions given by ANMs/ASHA workers.

### Study setting & participants

This study will be conducted in rural areas of Lucknow district, which is the capital of state of Uttar Pradesh in North India. Lucknow district has a population of 4,589,838, of which 33.79% are rural [6]. Here, there are 8 rural administrative blocks. Public health system for each of these blocks comprises of at least one (and in one block two) community health center (CHC) with outpatient care by doctors including pediatricians and 30 inpatient beds. Under each CHC are additional primary health centers (PHCs) for approximately 100,000 population with outpatient facilities and 4 beds. The lowest level of care is through a sub-center with an ANM. There is one subcenter for about 5 villages. Existing health infrastructure in Lucknow block at the initiation of the project is given in Table 1.

**Table 1:** Health Infrastructure of Lucknow Distric**t**

Health Infrastructure of Lucknow District	Number
Blocks	08
Community Health Centre (CHC)	09^a^
Total Sub Centre (SC)	345
Functional SC	331
Non Functional SC	14
Additional Primary Health Centre (APHC)	28
Accredited Social Health Activist (ASHA)	1246
Auxillary Nurse Midwife (ANM)	331
Super specialty hospitals	03
District combined hospitals	04
District hospitals	03
District Women hospitals	02
Number of AYUSH hospital/dispensary	78
Number of Ayurvedic hospital/dispensary	49
Number of homeopath hospital/dispensary	29

^a^BKT Block has two CHCs. Other 7 blocks have one CHC each

In the study, target population will be caregivers within the family of children < 5 years who were residing in the study area. No caregiver with a child < 5 years will be excluded.

### Study design

**Fig. 1:**
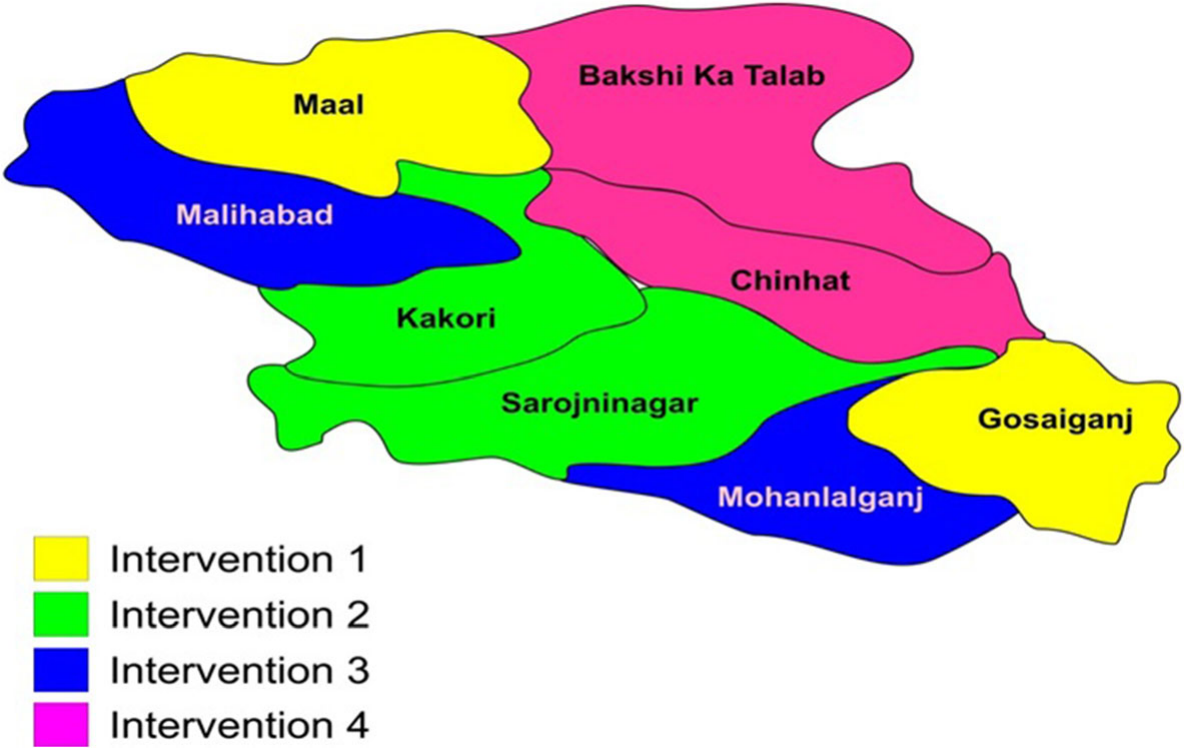
Block wise distribution of four project interventions

**Fig. 2:**
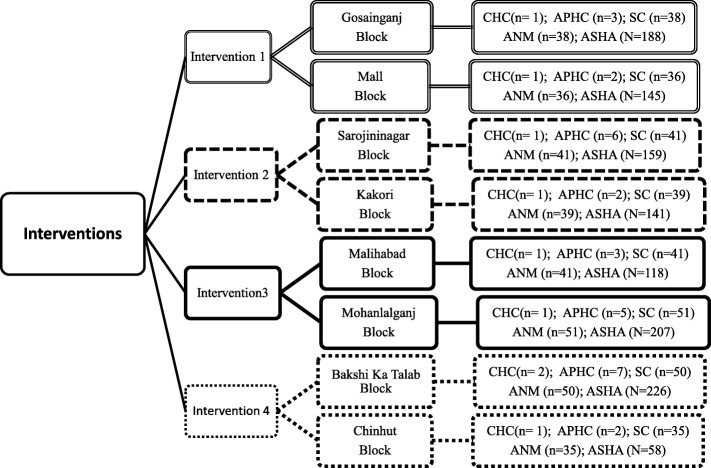
Distribution of rural health infrastructure across 8 blocks and their allocation to project intervention arms. `community orientation` CHC: Community Health Center; APHC: Additional Primary Health Centre; SC: Subcentre; ANMs: Auxiliary Nurse Midwifery; ASHA: Accredited Social Health Activist

A Community Based Open Labeled Behavioral Trial conducted in 2 by 2 factorial design (Table 2) and geographic distribution of these areas is given (Fig. 1). Two blocks, proportionately equal in terms of number of ASHA workers (roughly equal to be number of villages) have been purposively paired and then randomly assigned to an interventional arm. Health infrastructure in each intervention arm is given in Fig. 2.

**Table 2:** Design of the project

		PAS at APHCs/CHCs	
		Yes	No
PAS on VHND PLUSVillage IEC	No	Gosaiganj Block and Mall Block (Intervention1)	Bakshi Ka Talaab Block and Chinhat Block (Intervention 4)
	Yes	Malihabad Block and Mohanlalganj Block (Intervention3)	Sarojininagar Block and Kakori Block (Intervention2)

*Abbreviations PAS* Pneumonia Awareness Session, *VHND* Village Health and Nutrition Day, *IEC* information education communication, *PHC* Primary Health Centre, *APHC* Additional Primary Health Centre, *CHC* Community Health Center

#### Rationale for intervention

##### Establishment of PMU, PMC, PDK plus community orientation of doctors, ANMs and ASHA workers

Strengthening the capacity CHCs, PHCs and SCs for the management of CAP will result in better delivery of pneumonia specific care; this will build community’s trust in the public health system.

##### Behavior change communication for demand generation for pneumonia management by the community

To ensure optimal utilization of augmented health facilities by measures mentioned above, a behavior change in the community will be needed, with respect to management of CAP. This behavior change can be brought about by various BCC strategies that utilize the messages developed by us. Effective behavior change is likely to result in demand generation for better quality of care from the public health sector for CAP by the community.

To identify the most effective BCC strategy, PAS will be conducted for caregivers who voluntarily bring their children for immunization either at the PHC/CHC or on VHND at the *anganwadi* center (AWC) as are likely to be receptive to health education messages. There will also be diffusion of messages in the community. PAS will bridge the gap and build confidence of the community in the public health system and services. We will be able to identify what is the minimum effective package of services that will result in optimal utilization of augmented public health facilities.

#### Trainings

##### Orientation of doctors, ANMs and on ASHA workers on prevalent community pneumonia management practices

The participants will be given the innovative `community orientation` to CAP using vignettes of real life cases of CAP, informed about community barriers to case management and their perceptions of health facilities. Thereafter, they will be shown the messages developed and told about the rationale behind each. They will be told about infrastructural strengthening, namely, PDK, PMC, PMU (Table 3). Additional training will be given to the care providers separately as given below.

**Table 3:** Framework for Infrastructural Strengthening

Site	Infrastructural strengthening and purpose
Subcentre/PHC/CHC	PDK containing dispersible pediatric amoxicillin tablets (250 mg) PLUS instruction card will be provided by the project. Ten doses of amoxicillin (for a maximum of 5 days) will be kept in transparent envelop with *green sticker* for children below between 1 and 12 months and * yellow sticker* for children between 12 and 59 months. Each envelope will also have 4 tablets of paracetemol (500 mg). Within each envelope will have a small card with instructions for use of medicines on one side, and how to monitor a child with clinical pneumonia for improvement and deterioration on the other side will be kept. Instructions will be in Hindi.
APHC	PMC to treat pneumonia with fast breathing and stabilize &refer pneumonia with lower chest in-drawing with hypoxia (pulse-oximetry< 92%) and severe pneumonia. Project to provide: PDK, Pulse oximeter, spacer with baby mask, salbutamol inhaler. State Government Supply: Earmark ONE existing bed as *pediatric pneumonia bed,* storage facility for drugs, equipment and patient records and common facilities like 24X7 doctor-nurse; Injectibles: ampicillin, gentamycin, ceftrioxone, corticosteroid; Oral: antipyretics, co-trimoxazole; oxygen, face mask, suction machine and mucus extractors, laryngoscope, endotracheal-tube, ambu-bag, thermometer, pediatric blood pressure instrument.
CHC	PMU to treat pneumonia with fast breathing and lower chest in-drawing and admit pneumonia with hypoxia (pulse-oximetry< 92%) and severe pneumonia. Project to provide: PDK, Pulse oximeter, spacer with baby mask, salbutamol inhaler. State Government Supply: Earmark TWO existing beds as *pediatric pneumonia beds,* storage facility for drugs, equipment and patient records and common facilities like 24X7 doctor-nurse; Injectibles: ampicillin, gentamycin, ceftrioxone, corticosteroid and vasopressors (dopamine), calcium, potassium, intravenous fluids like dextrose saline, Ringer Lactate; oral: antipyretics, co-trimoxazole; bronchodilators; oxygen, face mask,suction machine and mucus extractors, laryngoscope, endotracheal-tube, ambu-bag, thermometer, pediatric blood pressure instrument, nebulizer with nebulizer solutions of salbutamol, epinephrine and steroid, intravenous cannula, venous cut-open set, heater/warm air blowers for winter months.

*Abbreviations APHC* Additional Primary Health Centre, *PHC* Primary Health Centre, *CHC* Community Health Center, *PDK* Pneumonia Drug Kit, *PMC* Pneumonia Management Corner, *PMU* Pneumonia Management Unit

A brief refresher course on ARI module of F-IMNCI will be organized for doctors. Medical management of CAP in paediatric ward of a tertiary care teaching hospital will be demonstrated. Doctors will also be trained to record clinical data of CAP patients in simple to use case sheet prepared for the project. Training will be done in KGMU by F-IMNCI trained faculty.

A brief refresher course on ARI module will be also be organized for the ANMs in their respective CHCs. Investigators and faculty trained in F-IMNCI will impart training. ANMs will be trained to use drugs from PDK in the villages and SC and document it. They will also be trained to refer CAP with lower chest in-drawing or severe pneumonia by calling ambulance services by dialing 102/108. ANMs will be trained to conduct PAS using (a) case-stories (in video/text formats) (b) messages for early recognition of pneumonia, when and where to seek care, risk perception of delayed treatment or treatment from unqualified provider and also for recognition of the danger signs of pneumonia through the self-developed posters, audio and video messages as well as (c) inform the community about infrastructural strengthening, PDK, PMC, PMU and (d) respond to queries.

ASHA workers will be provided the same training capsule as for the ANMs with the exception that (a) on finding a suspected case of CAP, ASHA workers will either contact the local ANM for immediate urgent case confirmation and treatment or expedite referral by calling ambulance services by dialing 102/108 (b) they will not dispense drugs from PDK (c) They will conduct PAS sessions in VHND using only with the cases stories and messages in text (poster) format. Project Staff at CHC will train them in batches. Table 3 provides the framework of infrastructural strengthening.

#### Medicines

For ambulatory care, oral amoxicillin DT (250 mg) will be used as recommended by the World Health organization for low HIV prevalence areas [7], Lucknow District being one of them. Amoxicillin DT (250 mg) will be packaged as PDK. PDK will be in platic zip locked 3 by 6 cm bags with either a green sticker (indicating use in children less than 12 months of age) or yellow sticker (indicating use in children between 12 and 59 months of age). PDK will have amoxicillin for a 5 day course + 2 additional doses for wastage (10 + 2 tablets `Green Kit` and 20 + 4 in `Yellow Kit`) (Table 4). Each kit will also have tablet paracetemol, instructions for use of medicines in Hindi, danger signs of pneumonia in Hindi and a card to mark number of days/doses per day of amoxicillin DT given. Each month ANM will be given 2 green and 2 yellow PDK through the CHC. Each CHC will get 25 PDKs/month and PHC will get 10 PDK/month. We will telephonically monitor the use of PDK and replenish them as and when needed to ensure uninterrupted supply.

**Table 4:** WHO’s New Pneumonia Treatment Guidelines for Community Case Management

Age	Pneumonia in low HIV Prevalence areas	Pneumonia in high HIV Prevalence areas	Severe Pneumonia	Severe Pneumonia Danger Signs
2–12 Months 4-10 kg	1 Amoxicillin 250 mg tablet/twice a day/3 days	1 Amoxicillin 250 mg tablet/twice a day/5 days	1 Amoxicillin 250 mg tablet/twice a day/5 days	1st dose antibiotic, referral to health facility for supportive therapy
12–59 Months 10-19 kg	2 Amoxicillin 250 mg tablets/twice a day/3 days	2 Amoxicillin 250 mg tablet/twice a day/5 days	2 Amoxicillin 250 mg tablets/twice a day/5 days	1st dose antibiotic, referral to health facility for supportive therapy

*Source:* UNICEF. Amoxicillin Dispersible Tablets (DT): Product Profile, Availability and Guidance (July2014).Assessed:http://www.unicef.org/supply/files/Amoxicillin_DT_Product_Profile_and_Supply_Update.pdf

In this project Amoxicillin tablets will be procured from Indian based company which has certificate of being compliant with manufacturing standards recommended by the World Health Organization [8]. For the treatment of severe pneumonia, as recommended by F-IMNCI [9], Injectable Ampicillin and gentamycin (or third generation cephalosporin as second line of treatment) will be used which is available at the APHC/CHC. If they need additional supplies, it will be procured and supplied through the project.

#### Process

##### Pre-intervention phase (6 months)

Standard operating procedures, training modules and data collection tools will be developed. Supplies (including drugs) will be procured. Working closely with the government, PMC and PMU will be established in APHC and CHC, respectively. Health staff will be trained. Baseline line Survey will be conducted in intervention and control areas to assess the burden of Acute Respiratory Illness (ARI)/CAP in under-5 children, care seeking pattern and behavior, socio-demographic conditions of households, health infrastructure and skills of service providers (KAP) for management of ARI/CAP. Survey will be carried out using population proportion sampling using 30 cluster methodology [10]. In all 2400 households will be selected from 240 villages.

#### Intervention phase (12 months)

Interventions that will be administered with existing health system have been described above. Time schedule is given as Additional file 1.

Description of Interventions is as follows:

*Intervention 1:* Organizing PAS using self-developed and validated IEC materials in PHCs and CHC monthly, conducted by a trained ANM (not involved with immunization) and project facilitator during routine immunization day. On this day about 30–50 parents with children come voluntarily at the CHC and APHC. The doctors at the APHC/CHC are also present at that time and besides supervising immunization will also give information to build awareness about pneumonia, if approached by the parents. A second ANM, not involved with immunization, will conduct IEC sessions in a separate corner of the immunization room or waiting area when a group of 10–15 caregivers have assembled.

During the PAS session, the ANM will use (a) case-stories (in video/text formats), (b) messages for early recognition of pneumonia, when and where to seek care, risk perception of delayed treatment or treatment from unqualified provider and also recognition of danger signs of pneumonia through the self-developed posters, audio and video messages as well as (c) inform the community about infrastructural strengthening, PDK, PMC, PMU and (d) respond to queries.

Dates for the PAS during immunization days will be fixed in advance with the administrative authorities. ASHA workers and ANMs will disseminate the date in their respective areas by word of mouth, through Gram Pradhan and Anganwadi Worker and mobilize the community.

The project staff will document the PAS proceedings, noting the number of persons who attended a particular session. The queries asked by attendees will be noted and over time a question and answer book will be prepared. On-site visits and telephonic contact will be made to validate conduction of PAS sessions. The project staff will conduct exit interviews of about 10% of the attendees noting their understanding of the materials explained to them and their satisfaction, using a pre-developed open-ended questionnaire (qualitative research methodology).

*Intervention 2:* PAS will be conducted during V.H.N.D. monthly by the ASHA worker for caregivers who congregate there using (a) case-stories (text formats) (b) messages for early recognition of pneumonia, when and where to seek care, risk perception of delayed treatment or treatment from unqualified provider and also recognition of danger signs of pneumonia through the self-developed posters, as well as (c) inform the community about infrastructural strengthening, PDK, PMC, PMU and (d) respond to queries.

*Intervention 3:* Combination of Both Intervention 1 & 2:

*Intervention 4:* This will be the Usual Care arm. In this arm only PDK, PMC and PMU will be established. No IEC will be done in the villages or APHC/CHC. Children in in the usual arm group will receive the same standard care and services provided to all children and their families residing in the area.

##### Quarterly health facility audit

This will be done to collect data on process Indicators and will be used for the establishment of Management information-system. Process indicators will be (i) utilization of PDK, PMC and (ii) conduct of PAS sessions in APHC, CHC and during VHND as well as numbers attending it. This will be done by the project staff. Data will be abstracted from records of PHC/CHC and SC for number of clinical pneumonia treated either as outpatients or inpatients or referred (with reasons and place) in last 1 month, and utilization of PDK and availability of medicines and equipment for the treatment of CAP (both provided through the project and supplied from the government). This information will be collated and shared with the Medical officer-In Charge of the health facility, Chief Medical Officer of Lucknow and office of Mission Director, National Health Mission.

#### Post intervention phase (6 months)

Post-intervention, primary outcome measures, e.g. utilization of public facility for management of CAP will be assessed through health facility audit. End line survey, similar to baseline survey, will be conducted to measure the changes due to proposed intervention in community’s preference of health providers for treatment of ARI/CAP. Data management, analysis and report writing will be done. Results will be widely disseminated. For widespread dissemination, study protocol and findings will be published in indexed peer-reviewed journals. Technology transfer will be done to the state government to scale up establishment of PMC, PMU and provide PDK plus implementing the most effective behavior change communication strategy for the entire state. Data will be accessible to public researchers after the study findings have been published.

### Data management & analysis plan

Data will be collected in pre-designed questionnaires preferably using electronic data collection system. Data quality assurance techniques and data cleaning procedures will be deployed before final analysis. Data will be analyzed using SPSS version 18 (Chicago, IL). Since this is a behavioral trial and no pharmacological intervention is being given, we do not plan to perform interim analysis.

Univariate distribution of baseline and outcome variables would be assessed by frequency counts. Outliers will be identified, reported and excluded from analysis if required. They would be compared between interventions using chi square test for categorical and Student’s t test and ANOVA for continuous variables. A *p* value of < 0.05 will be taken as statistically significant, using a two tailed distribution. Adherence to the intervention across each arm will be calculated and compared across the three arms (as one was a control arm). Good intervention is defined as > 75% adherence to the sessions in the duration of the project. Primary outcomes will be number of PDK kits distributed to children plus the number of children treated by government functionaries for acute respiratory infection (ARI) or pneumonia without PDK, using medicines available at the health facility in interventions given by ANMs/ASHA workers. If we find that the diagnosis of children being treated is not mentioned, then we fill this missing information by extrapolation from data where diagnosis is given for the same month and within the intervention arm, assuming that there would be similar proportion of cases with this diagnosis. We will compute the difference in the proportion of ARI/pneumonia treated in each intervention arm when compared to control arm in the intention to treat analysis. For the secondary outcome, we will compare the proportion of cases with ARI/pneumonia treated by government providers as their first choice in interventions given by ANMs/ASHA workers, as an intention to treat analysis. In the per-protocol analysis we will compare the proportion of cases with ARI/pneumonia treated by government providers as their first choice in interventions given by ANMs/ASHAs from those households which have participated in the baseline as well end line survey using paired t-test. As a sub-analysis, we will compare the mean, median and interquartile range of out of pocket expenditure for ARI/pneumonia in the baseline and end line survey within each arm. As a comparator, we will do similar analysis for cases who have suffered from diarrhea to assess whether changes in spending were a function of time or because of care seeking from government providers, which is most inexpensive.

A qualitative narrative of the process of establishment of the project will be given. Key informant and semi-structured interviews will be used to assess the level of satisfaction of stakeholders with (a) the services provided by public health facility augmentations and (b) IEC campaign strategies. Qualitative analysis techniques will be used for this data analysis.

Primary outcomes will be (a) number of patients of CAP treated by ANMs/doctors with medicines from PDK OR treated PMC OR PMU and (b) health service providers’ preference for treatment of CAP/ARI. For health facility utilization, the data will be abstracted periodically from the records maintained there. Feedback will be given to each facility on the process indicators within a month. For health provider preference, data from base line and end-line multistage cluster surveys will be used. At the end of the project the primary outcome measures will be compared in interventions given by ANMs/ASHA workers, using tests of proportion.

#### Process indicators

- Capacity building of health staff through trainings at the initiation of process and retraining after one year. Proportion of doctors, ANMs and ASHAs trained by each intervention block will be computed.
- Establishment of PMC and PMC: For this we will request the government to pass relevant orders. Project and government will provide materials as given in Table 3.
- Distribution of PDK: Project will purchase and repackage the medicines and distribute them at various health facilities. From here PKD will be given to the ANMs also. This will be monitored by the project.
- Utilization of PDK: Project will monitor the number of PKDs distributed at each quarter in each block.
- Utilization of PMC and PMU: This will be assessed by number of cases of CAP admitted at each facility by intervention type.
- Conduct of BCC interventions: This will be verified by project staff continuously throughout the project. Quality assessment of the PAS sessions will also be done by taking feedback from a convenience sample of attendees.

### Adverse events monitoring

Approximately 5% of caregivers of cases of CAP who have received PKD will be contacted. They will be asked about their perceptions of the medicines and cards provided in the kit, whether the child required hospitalization or improved after taking medicines and if the child had diarrhea, rash, vomiting or any other complaints. This will be done by visiting their homes.

### Ethics and research governance

This study (protocol version 2 dated 19.12.2015) has been approved by the Health Ministry Steering Committee of the Indian Council of Medical Research, New Delhi (India), the Institutional Ethics Committee, King George’s Medical University (KGMU), Lucknow (India) and relevant public authorities of state government. Written informed consent will be obtained by the project staff from all participants. To protect confidentiality of respondents, their identifiers will not be noted in any data collection instrument. Visit Log books, questionnaires, and project documents will be stored in a locked area, not accessible to unauthorized individuals.

Technical Advisory Group (TAG) for the project will be constituted to ensure quality control and government buy-in for the research findings. Members from government and non-government sectors, civil society organizations, grass root workers, academia and sponsors will be part of TAG. This group will meet twice, first at the time of project initiation and then immediately before project completion.

To ensure that the entire community of the district of Lucknow is benefitted through improved delivery of pneumonia specific health care by the public health system, PMU, PMC and PMKs will be provided to all the CHCs, APHCs, and SC of Lucknow District. This will ensure equity and distributive justice. However, there is doubt about the best way of community mobilization. The availability of better care facilities alone may drive improved care seeking for CAP. If that is the case, we have a usual care arm in our behavioral trial. If this is not the case, we will try out three other interventions, first public health facility based (CHC/APHC), second village based and the third a combination of both. The most effective strategy for improved care seeking of CAP (as a result of behavior change) will be identified and expanded into the entire district.

## Discussion

CAP is one of the leading killers of childhood deaths worldwide. An estimated 2 million deaths occur yearly due to community-acquired pneumonia (CAP) in children < 5 [1]. Among these about half a million die in India. Every year, approximately 43 million new cases of pediatric pneumonia are reported in India [11]. Poor and delayed care-seeking has been implicated in 6–70% of child deaths in developing countries, including those from pneumonia [12–14].

In the setting of this project we found in an earlier work that vernacular term “pneumonia” was mentioned by most caregivers regardless of age without prompting, indicating that the term had entered popular health culture. We found that recognition of pneumonia and its danger signs were poor among caregivers. In addition, it was found that fast breathing, an early sign of pneumonia, was not commonly recognized and chest in-drawing though recognized was not commonly monitored by removing a child’s clothing [3]. Limited recognition of fast breathing and chest in drawing- two key signs of pneumonia has been reported in many other studies [15–18].

As a part of an earlier work, we also found in Lucknow district that recognition of danger signs of pneumonia was poor among caregivers [19]. Caregivers reported symptoms like fever, cold, coughing as danger signs much more than Integrated Management of Neonatal and Childhood Illness (IMNCI) Danger Signs. A study conducted in Guatemala found that families are much more likely to visit a health care provider when their child experiences fever and gastrointestinal symptoms than when suffering from respiratory and other symptoms [20]. Another study in Nairobi slums reported that care-seeking from medical providers was significantly higher for diarrhea than for ARI [21]. We also found in our study that even after the disease was recognized there was a delay in seeking treatment [22].

In Lucknow district as in other districts of Uttar Pradesh and Bihar most cases CAP are taken to village-based, mostly unqualified, rural medical practitioners (RMP), and when condition deteriorates children are rushed to private clinics in towns nearby. Reasons cited for the preference of RMP included their ready availability, easy accessibility, the fact that it was culturally acceptable for women to consult these local practitioners unaccompanied by their husbands, low fees and availability of credit [3]. Systematic Review on Care-seeking practices in South Asia [23] provided evidence that families preferred remedies from traditional healers rather than skilled health workers because of cultural and religious beliefs, poor access to health facilities, and financial barriers. A study conducted in Egypt found that even though mothers were able to recognize pneumonia signs but they did not use this recognition for appropriate care-seeking [24].

In rural India RMPs are seen as appropriate doctors by caregivers, although they did not have professional training in allopathic medicine. Display and use of modern medical paraphernalia made caregivers believe that most treatment provided by RMPs results in a good outcome. Studies conducted in Northern India [3, 25, 26] and Southern India [27] also provide evidence that RMPs treat minor illnesses, provide first relief, refer patients to other providers and administer formally prescribed treatments and this makes them the first point of contact over qualified practitioners.

ASHAs, the frontline health functionaries for basic preventive care, also have an important role to play in CAP identification and referral to ANMs or higher-level public facilities. Community does seek information from the ASHAs on childhood illnesses, but ASHAs have limited knowledge about the signs of CAP and its management [3]. Even the ANMs did not have clear information on how to manage childhood pneumonia cases. Although the ANMs correctly knew how to monitor improvement/deterioration in CAP they did not feel competent enough to assess, classify and treat with minimum essential drugs before referral. The CHWs are being trained under Integrated Management of Childhood Illness (IMCI) to manage pneumonia sick child but poor supervision, inadequate essential supplies and lack of refresher trainings may affect the performance of these workers [28]. Studies conducted in other countries have, however, provided evidence that with appropriate training which emphasizes on pneumonia assessment, adequate supervision, and provision of drugs and necessary supplies, CHWs can significantly impact pneumonia specific mortality [29–32]. When we interviewed the CHWs even they were enthusiastic about learning more about CAP and participated in validation of educational messages developed by our team as a part of earlier project [3].

Community preferred health care seeking from private health facilities as compared to the government [22]. Negative perceptions of government medical facilities were related to unavailability/limited availability of necessary medicines and diagnostic tests, the perception that medicines available were of poor quality, overcrowding and referral of critical patients to distant government hospitals. A study conducted in Haryana, India explored reasons for underutilization of government health facilities. Reasons cited included lack of quality care, abominable behaviour of hospital staff, poor transportation facilities, and frequent referrals to higher centres [33]. A Nigerian study also found government facilities to be poorly managed leading to their underutilization [34]. It has been found that inadequacy in the quality of child health services in PHC facilities is a product of failures in a range of quality measures: structural i.e. lack of equipment and essential drugs and process failings i.e. non-use of the national case management algorithm and lack of a protocol of systematic supervision of health workers [35]. These structural and process factors need to be addressed so that the public health facilities are able to deliver effective services.

Many of the childhood deaths due to CAP can be averted by creating awareness in the community about signs and symptoms of pneumonia and the risk associated with it, as well as informing them about appropriate and timely care seeking. Community case management for pneumonia has associated with a 32% reduction in pneumonia specific mortality. For pneumonia, community interventions increased the care seeking behavior by 13% and the treatment failure rates also reduced by 40% [36]. Others have also shown that community interventions which are viable, effective and practical can have a sustainable impact on pneumonia specific mortality [37, 38] and neonatal mortality [39].

UNICEF, the World Health Organization (WHO) and their technical partners, developed IMCI strategy for the integrated management of five most important causes of childhood deaths including pneumonia [40]. The essential pillars of IMCI include improvement in the case management skills of health personnel, improvement in health systems, and improvement in family and community practices [41]. A study in Peru proposed informative printed media and audio-visual kits in waiting rooms of health establishments, or community education programs such as socio-drama to improve family and community practices [42]. In addition to this, Mathew JL et al. 2011 stressed the need to leverage gap in utilization of existing government health services for childhood pneumonia [43]. Therefore, we suggest that building confidence in government health staff for treating and triaging cases of CAP, possibly by timely and appropriate referral and setting up dedicated round the clock “pneumonia care units/corners” in government hospitals is urgently required.

We therefore propose this study, which assesses the effectiveness of various communication strategies for improving childhood pneumonia case management interventions at the mother/community level, health worker and health center level.

This project work will be done in partnership with the state government. This will ensure effective execution of research work. Simultaneously, capacity building of the public health staff of Lucknow district will be done. The doctors as well as ANMs/ASHA workers will be reoriented to pneumonia management plus be with community’s perception about recognition and care seeking for CAP and reasons for not opting to bring their child to public health facility as a first choice. This has not been done so far in any government program. This will ensure their emotional motivation and commitment to fight pneumonia by giving their best efforts. The project will generate demand and improve supply of quality of care of CAP and thus result in reduced mortality in Lucknow district. Since the work will be done in partnership with the government, it can be scaled up.

## Additional file

Additional file 1: Schematic diagram of time schedule (DOC 55 kb)

## Abbreviations

ANMs: Auxiliary Nurse Midwifery
APHC: Additional Primary Health Centre
ARI: Acute Respiratory Illness
ASHA: Accredited Social Health Activist
AWC: Anganwadi centre
AWW: Anganwadi worker
BCC: Behavior change communication
CAP: Community Acquired Pneumonia
CHC: Community Health Center
IEC: Information, Education and Communication
NHM: National Health Mission
PAS: Pneumonia Awareness Session
PDK: Pneumonia Drug Kit
PHC: Primary Health Centre
PMC: Pneumonia Management Corner
PMU: Pneumonia Management Unit
SC: Sub centre
V.H.N.D.: Village Health and Nutrition Day
