# Supplementary material for: To assess the effectiveness of various communication strategies for improving childhood pneumonia case management: study protocol of a community based behavioral open labeled trial in rural Lucknow, Uttar Pradesh, India
Source: BMC Pediatr. 2018 Aug 22;18:279. doi: 10.1186/s12887-018-1250-4 (PMC6106877; doi:10.1186/s12887-018-1250-4)
Supplement: Supplementary file 1 — Schematic diagram of time schedule (DOC 55 kb) [file 12887_2018_1250_MOESM1_ESM.doc]

Figure 1. Schedule of enrolment, interventions, and assessments

|  | **STUDY PERIOD** | | | |
| --- | --- | --- | --- | --- |
|  | **Enrolment** | **Allocation** | **Post-allocation** | **Close-out** |
| **TIMEPOINT** | ***-t_1_*** | **0** | ***Time Period*** | ***t_x_*** |
| **ENROLMENT** |  |  |  |  |
| *Eligibility screen* | Feb.2016 | × | × | May 2016 |
| *Informed consent* | Feb.2016 | × | × | May 2016 |
| **INTERVENTIONS** |  |  | October 2016 to December 2017 |  |
| *[Intervention 1]* | × | Sep. 2016 |  | Dec. 2016 |
| *[Intervention 2]* | × | Sep. 2016 |  | Dec. 2016 |
| *[Intervention 3]* | × | Sep. 2016 |  | Dec. 2016 |
| *[Intervention 4]* | × | Sep. 2016 |  | Dec. 2016 |
| **ASSESSMENTS** |  |  |  |  |
| 1. ***Baseline Variables*** |  |  |  |  |
| - *Socio-demographic* | Feb.2016 | × | × | September 2018 |
| - *Episodes of respiratory illnesses in children* | Feb.2016 | × | × | September 2018 |
| - *Hospitalization for possible community acquired pneumonia* | Feb.2016 | × | × | September 2018 |
| 1. ***Outcome variables*** | Feb.2016 | × | × | September 2018 |
| - *Health care seeking pattern for community acquired pneumonia specifically from government health facilities* | Feb.2016 | × | × | September 2018 |
| - *Utilization of Pneumonia Drug Kit* | Feb.2016 | × | × | September 2018 |
